# School tobacco-related curriculum and behavioral factors associated with cigarette smoking among school-going adolescents in Zambia: Results from the 2011 GYTS study

**DOI:** 10.18332/tid/146960

**Published:** 2022-05-05

**Authors:** Cosmas Zyambo, Pawel Olowski, David Mulenga, Franklin Liamba, Paul Syapiila, Seter Siziya

**Affiliations:** 1 Department of Community and Family Medicine, School of Public Health, University of Zambia, Lusaka, Zambia; 2 Department of Epidemiology and Biostatistics, School of Public Health, University of Zambia, Lusaka, Zambia; 3 Department of Clinical Sciences, Copperbelt University School of Medicine, Ndola, Zambia

**Keywords:** Zambia, adolescents, cigarette smoking, tobacco-related curriculum

## Abstract

**INTRODUCTION:**

Tobacco smoking is a global public health concern. It has been projected that children and young people who are alive today in developing countries will bear the most burden of tobacco-related morbidity and mortality in the near future. This study investigated the school tobacco-related curriculum and behavioral factors associated with cigarette smoking among school-going adolescents.

**METHODS:**

We accessed secondary data in a public domain collected using a cross-sectional study design. Altogether, 3377 seventh to ninth grade students were selected by stratified two-stage cluster sampling. Data were collected using a Global Youth Tobacco Survey (GYTS) Core Questionnaire. Multivariate logistic regression models were used to determine associations of school tobacco-related curriculum and behavioral factors with current cigarette smoking status. Adjusted odds ratios and their 95% confidence intervals are reported.

**RESULTS:**

Of the 2611students included in the analysis, 6.8% (7.8% of males and 5.8% of females) reported smoking cigarettes. Slightly over half of the students were taught in schools about the effects of smoking (53.6%) and the dangers of smoking (64.1%). Adolescents who had friends who smoked were more likely to smoke compared to those who did not have friends who smoked. Adolescents whose parents smoked were more likely to smoke compared to those who did not have parents who smoked. Adolescents who were not taught at school about the dangers of smoking, or were not sure about it, were more likely to smoke compared to those who were taught (AOR=1.94; 95% CI: 1.28–2.94).

**CONCLUSIONS:**

Schools play an important role in shaping smoking behavior among school-going adolescents. Based on our findings, school programs aimed at reducing cigarette smoking among school-going adolescents may achieve greater impact by implementing anti-smoking interventions that involve parents and peers in smoking prevention activities, and have a robust tobacco school curriculum.

## INTRODUCTION

Despite the large reduction in the global prevalence of daily tobacco smoking in the general population^1-3^, the number of the absolute smokers especially males have been rising significantly from 1.050 billion in 2000 to 1.093 billion in 2018^3,4^. More than 80% of the 1.3 billion smokers globally live in low- and middle-income countries, which are apparently the target of tobacco industry marketing^5^. According to the Global Youth Tobacco Surveillance (GYTS) special report by Warren et al.^6^, who did a cross-country comparison of tobacco use among youths in 43 countries, current use of tobacco products ranged from 3.3% to 62.8% and the current cigarette smoking ranged from less than 1% to 39.6%. Tobacco smoking is an addictive behavior sustained by addiction to nicotine^7,8^, tobacco use often starts in adolescence^9^, and as these adolescents become adults, they serve as role models for other youths, hence reinforcing the vicious cycle^10^. Their emotional instability, coupled with the rapid development of their bodies and the excessive pressures from society, predisposes them to be at risk of engaging in negative vices such as pre-marital sex, smoking, alcohol, and drug abuse among others^11^; and hence leading to poor performance in school^12^. There is substantial evidence to suggest that adolescents who smoke are also highly likely to engage in risky social behaviors such as alcohol abuse, illicit drug use, and pre-marital sex^11,13^.

Zambia ratified the World Health Organization, Framework Convention on Tobacco Control (WHO FCTC) in 2008, since then several policies have been implemented to reduce smoking not only in adults but in adolescent as well. The following are some of the restrictions imposed: prohibiting smoking in public places^14^; banning cigarette sales to minors who are aged <16 years and giving free tobacco products^15^; banning of tobacco advertisement in the mass media; and banning of smoking in educational facilities, healthcare facilities, public transport and other public places^15^. Apart from these regulations, the WHO recommended that adolescents have a tobacco-related school curriculum where the students would be taught the dangers of tobacco smoking. Despite the tobacco-related school curriculum implementation in Zambia, there has not been any significant reduction in adolescents’ cigarette smoking between 2002 and 2007 GTYS^16,17^.

The lack of improvement in smoking rates is possibly multifactorial however, amidst other interventions that have been put in place, the impact of the tobacco-related school curriculum on smoking prevention among adolescents remains unknown. No data have explored associations between school tobacco-related curriculum factors and cigarette smoking status among adolescents in Zambia. This study aims to explore these associations and the results will therefore inform policy and specific school-based anti-smoking programs on reducing cigarette smoking among adolescents.

## METHODS

### Data source

GYTS is a school-based survey of students, in grades 7 to 9, who are aged 11–18 years. It is designed to produce cross-sectional nationally representative estimates. GYTS methodology for constructing the sample frame, selecting schools and classes, and processing data is standardized. The survey uses a standard core questionnaire on tobacco use and key tobacco control indicators as well as allows adapting to the needs of the country. A more comprehensive description of the overall GYTS aims and methodology are available elsewhere^18^. The Center for Disease Control and Prevention (CDC) determined the sample size and sampled the schools using a standard protocol and software developed by CDC after receiving the sampling frame from the GYTS research coordinator. Although the actual sample size or how it was computed was not available, a minimum sample size of 1500 students of whom about half are female is required for GYTS surveys^19^. The overall response rate of the school-going student surveyed was 55.7%. A total of 3377 school-going students were surveyed; and of the 2648 students who answered the specific outcome smoking question, 37 (1.4%) were excluded from the analysis for missing variables; 2611 schoolgoing students were included in the final analysis. Given that the primary school enrollment rate is 98.7%^20^, this age group is representative of the total population of this age in Zambia.

### Dependent variable

The outcome variable of interest was the current cigarette smoking status that was derived from the question: ‘During the past 30 days (one month), on how many days did you smoke cigarettes?’ with response options: 0, 1–2, 3–5, 6–9, 10–19, 20–29, all 30 days. Adolescents who smoked at least once during the previous 30 days were classified as current smokers, those who did not were classified as non-current smokers.

### Independent variables

*Sociodemographic factors*

These were: age (<14, 14, 15, 16, and ≥17 years), sex (male and female), and grade (seven, eight and nine).

*Behavioral factors*

These were: closest friends smoke cigarettes (none, some, most, and all); and parents smoke cigarettes (none, both, father only and mother only).

*Curriculum factors*

Our study used four questions from the questionnaire. The first three questions the student answered ‘yes’, ‘no’ or ‘not sure’ to questions: 1) ‘During this school year, were you taught in any of your classes about the dangers of smoking?’; 2) ‘During this school year, did you discuss in any of your classes the reasons why people your age smoke?’; 3) ‘During this school year, were you taught in any of your classes about the effects of smoking like it makes your teeth yellow, causes wrinkles, or makes you smell bad?’; and 4) ‘How long ago did you last discuss smoking and health as part of a lesson?’. Students’ answers for this question were categorized as never, this term, two terms ago, three terms ago, or more than a year ago. In Zambia educational institutions such as primary school (grades 1–7) and secondary school (grades 8–12), use the term system, with three terms in an academic calendar year.

### Statistical analysis

Descriptive statistics were used to obtain numbers and proportions of current smokers and non-smokers by their sociodemographic characteristics, as well as school curriculum factors. The chi-squared test of association was used to determine associations between various factors and current smoking status. Furthermore, univariate and multivariate logistic regression models were generated to examine the associations between factors that may influence cigarette smoking among school-going adolescents in Zambia. Variables that were found to be significantly associated (p<0.05) with current cigarette smoking status in the univariate analyses were included in the multivariate models. Investigator-led stepwise backward regression was used to identify the final model through elimination from the full model of all the variables with the highest p-values one at a time until all remaining model variables were significant (p<0.05). Post-estimation analysis using Akaike’s information criterion and Bayesian information criterion was used to compare the full and final (nested) model to confirm the best-fit model. Adjusted odds ratios (AORs), p-values, and the associated 95% confidence intervals (CIs) were estimated and used as measures of magnitude of associations. Age, gender, closest friends’ smoke cigarettes, parents smoke, taught at school about the dangers of smoking, and last discussion at school about smoking and health were controlled for in the full model. The analysis has not been weighted due to the poor response rate. Subsequently, a weighting factor was not applied to each student record to adjust for non-response, as well as a post-stratification adjustment by gender and grade was not done due to lack of required population frequencies and weights data for the GYTS sample. All analyses were conducted using STATA version 15.

## RESULTS

This study involved 2611 school-going adolescents and the overall prevalence of smoking cigarettes was 6.8% (7.8% of males and 5.8% of females). The largest proportion among adolescents who smoked cigarettes was found in males aged ≥17 years (30.6%) and in those who attended ninth grade (39.2%) (Table 1). Slightly over half of the students were taught in schools about the effects of smoking (53.6%) and dangers of smoking (64.1%) and, overall, smoking was significantly associated at the p<0.05 level with all sociodemographic, behavioral, and some school curriculum factors, except grade, being taught at school about smoking effects and having discussed at school the reasons why young people smoke.

**Table 1:** The characteristics of school-going adolescents in Zambia according to their sex and smoking status using Global Youth Tobacco Survey 2011 (N=2611)

*Characteristics*	*Males (1282; 49.1%)*		*Females (1329; 50.9%)*		*Overall*
	*Current smokers*		*Current smokers*		
	*Yes*	*No*	*Yes*	*No*
	*n (%)*	*n (%)*	*n (%)*	*n (%)*	*n (%)*
**Total**	100 (7.8)	1182 (92.2)	77 (5.8)	1252 (94.2)	2611 (100)
**Age** (years)					
<14	12 (12.2)	276 (23.6)	15 (15.9)	358 (28.7)	661 (25.5)
14	16 (16.3)	238 (20.3)	26 (24.0)	300 (24.1)	580 (22.4)
15	21 (21.4)	240 (20.5)	13 (19.7)	285 (22.9)	559 (21.6)
16	19 (19.3)	234 (20.0)	17 (19.7)	192 (15.4)	462 (17.8)
≥17	30 (30.6)	182 (15.6)	6 (20.8)	111 (8.9)	329 (12.7)
**Grade**					
Seven	23 (23.7)	421 (36.0)	31 (41.9)	447 (36.1)	922 (35.7)
Eight	36 (37.1)	384 (32.9)	26 (35.1)	433 (34.9)	879 (34.1)
Nine	38 (39.2)	363 (31.1)	17 (23.0)	360 (29.0)	778 (30.2)
**Closest friends smoke cigarettes**					
None	26 (26.5)	830 (71.2)	24 (31.6)	917 (74.5)	1797 (69.9)
Some	41 (41.8)	232 (19.9)	36 (47.3)	226 (18.3)	535 (20.8)
Most	17 (17.4)	68 (5.8)	12 (15.8)	51 (4.1)	148 (5.8)
All	14 (14.3)	35 (3.1)	4 (5.3)	38 (3.1)	91 (3.5)
**Parents smoke**					
None	46 (54.8)	800 (81.2)	21 (32.8)	856 (81.8)	1723 (79.0)
Both	6 (7.1)	13 (1.4)	8 (12.5)	27 (2.6)	54 (2.5)
Father only	28 (33.3)	157 (15.9)	21 (32.8)	157 (14.9)	363 (16.7)
Mother only	4 (4.8)	15 (1.5)	14 (21.9)	7 (0.7)	40 (1.8)
**Taught at school about the dangers of smoking**					
Yes	56 (57.7)	742 (64.3)	31 (40.8)	804 (66.3)	1633 (64.3)
No or not sure	41 (42.3)	412 (35.7)	45 (59.2)	409 (33.7)	907 (35.7)
**Discussed at school the reasons why young people smoke**					
Yes	32 (37.7)	513 (45.4)	27 (40.9)	541 (44.9)	1113 (44.8)
No or not sure	53 (62.3)	616 (54.6)	39 (59.1)	663 (55.1)	1371 (55.2)
**Taught at school about smoking effects**					
Yes	49 (52.1)	627 (55.1)	30 (42.3)	643 (52.8)	1349 (53.5)
No or not sure	45 (47.9)	511 (44.9)	41 (57.7)	574 (47.2)	1171 (46.5)
**Last discussion at school about smoking and health**					
Never	22 (22.7)	504 (43.9)	19 (25.0)	471 (38.8)	1016 (40.1)
This term	23 (23.8)	279 (24.3)	24 (31.6)	342 (28.2)	668 (26.4)
Two terms ago	17 (17.5)	130 (11.3)	10 (13.1)	157 (12.9)	314 (12.4)
Three terms ago	11 (11.3)	66 (5.8)	7 (9.2)	51 (4.2)	135 (5.3)
More than a year ago	24 (24.7)	168 (14.7)	16 (21.1)	193 (15.9)	401 (15.8)

All variables were significant at the p<0.05 level except for: grade; taught at school about smoking effects; and discussed at school the reasons why young people smoke. Missing values for the total respondents: age 0.8% (20); grade 1.2% (32); closest friends smoke cigarettes 1.5% (40); parents smoking 16.5% (431); taught at school about the dangers of smoking 2.7% (71); discussed at school the reasons why young people smoke 4.9% (127); taught at school about smoking effects 3.5% (91); and last discussion at school about smoking and health 2.9% (77).

Results from the bivariate logistic regression analyses illustrate that the following factors were statistically associated with current cigarette smoking status among school-going adolescents in Zambia: age, grade, friends’ cigarette smoking status, parents smoking status, being taught at school about the dangers of smoking, and having a discussion at school about smoking and health (Table 2). Multivariate logistic regression analysis was used to obtain adjusted estimates for current cigarette smoking status given the various independent variables. School-going adolescents who had some of the closest friends who smoked, most of the friends who smoked and those who had all friends who smoked, were more likely to smoke compared to those who did not have friends who smoked cigarettes (AOR=3.93; 95% CI: 2.51–6.15, AOR=8.35; 95% CI: 4.47–15.59, and AOR=5.92; 95% CI: 2.72–12.88, respectively). Adolescents whose parents smoked were more likely to smoke compared to those with parents who did not smoke, with the highest odds in the case of a mother only followed by both parents smoking (AOR=10.13; 95% CI: 4.37–23.47, and AOR=6.53; 95% CI: 3.12–13.66, respectively). Adolescents who were not taught at school about the dangers of smoking or were not sure about it were more likely to smoke compared to those who were taught (AOR=1.94; 95% CI: 1.28–2.94). Adolescents who had a discussion at school about smoking and health in the current term, term 2, term 3, or more than a year ago, had higher odds of smoking compared to those who never had such a discussion (AOR=2.89; 95% CI: 1.64–5.10, AOR=3.85; 95% CI: 2.00–7.39, AOR=7.09; 95% CI: 3.27–15.40, and AOR=3.64; 95% CI: 1.97–6.74, respectively).

**Table 2:** Factors associated with current cigarette smoking status among school-going adolescents in Zambia, Global Youth Tobacco Survey (GYTS) 2011 (N=2611)

*Factors*	*OR (95% CI)*	*p*	*AOR (95% CI)*	*p*
**Age** (years)				
<14 (Ref.)	1		1	
14	1.80 (1.11–2.92)	**0.017**	1.94 (1.05–3.56)	**0.033**
15	1.51 (0.92–2.50)	0.106	1.16 (0.61–2.22)	0.652
16	1.86 (1.12–3.07)	**0.016**	1.95 (1.02–3.71)	**0.042**
≥17	2.86 (1.73–4.73)	**<0.001**	2.48 (1.30–4.71)	**0.006**
**Sex**				
Male (Ref.)	1		1	
Female	0.73 (0.53–0.99)	**0.042**	0.80 (0.54–1.19)	0.272
**Grade**				
Seven (Ref.)	1			
Eight	1.21 (0.84–1.75)	0.296	NA	
Nine	1.15 (0.79–1.69)	0.467		
**Closest friends smoke cigarettes**				
None (Ref.)	1		1	
Some	5.56 (3.87–8.00)	**<0.001**	3.93 (2.51–6.15)	**<0.001**
Most	8.56 (5.29–13.83)	**<0.001**	8.35 (4.47–15.59)	**<0.001**
All	8.17 (4.62–14.48)	**<0.001**	5.92 (2.72–12.88)	**<0.001**
**Parents smoke**				
None (Ref.)	1		1	
Both	8.97 (4.73–17.00)	**<0.001**	6.53 (3.12–13.66)	**<0.001**
Father only	3.85 (2.63–5.63)	**<0.001**	2.41 (1.55–3.75)	**<0.001**
Mother only	18.71 (9.66–36.27)	**<0.001**	10.13 (4.37–23.47)	**<0.001**
**Taught at school about the dangers of smoking**				
Yes (Ref.)	1		1	
No or not sure	1.89 (1.40–2.56)	**<0.001**	1.94 (1.28–2.94)	**0.002**
**Discussed at school the reasons why young people smoke**				
Yes (Ref.)	1		NA	
No or not sure	1.34 (0.96–1.87)	0.084		
**Taught at school about smoking effects**				
Yes (Ref.)	1		NA	
No or not sure	1.29 (0.95–1.76)	0.105		
**Last discussion at school about smoking and health**				
Never (Ref.)	1		1	
This term	1.79 (1.18–2.72)	**0.006**	2.89 (1.64–5.10)	**<0.001**
Two terms ago	2.19 (1.34–3.58)	**0.002**	3.85 (2.00–7.39)	**<0.001**
Three terms ago	3.43 (1.92–6.13)	**<0.001**	7.09 (3.27–15.40)	**<0.001**
More than a year ago	2.52 (1.62–3.92)	**<0.001**	3.64 (1.97–6.74)	**<0.001**

NA: variables excluded from the multivariable analysis due to lack of statistical significance in univariate analysis: grade, discussed at school the reasons why young people smoke, and taught at school about smoking effects. AOR: adjusted odds ratio; adjusted for age group, gender, closest friends’ smoke cigarettes, parents smoke, taught at school about the dangers of smoking and last discussion at school about smoking and health.

## DISCUSSION

To the best of our knowledge, this is the first report that assesses tobacco-related school curriculum and adolescents’ smoking behavior using the GTYS in Zambia. Overall, slightly half of the students were taught in schools about the effects of smoking (53.6%) and the dangers of smoking (64.1%). In our study, 6.8% of school-going adolescents reported current cigarette smoking. This estimate is lower than what has been reported in previous studies among school-going adolescents both in Zambia and other countries^21-23^, and yet remains higher than the prevalence in other countries^23-25^. It is, however, interesting to note that the school-going adolescents smoke cigarettes far much less than they smoke other tobacco products in major cities in Zambia^26^. In the capital city Lusaka alone, in 2002 and 2007 GYTS, 9.2% and 6.8% of the students reported to be current cigarette smokers, respectively, while 17.7% and 22.8% reported smoking other tobacco products, respectively^26^. The explanation could be that due to the taxes, prices and ban on accessing cigarettes, adolescents have opted to smoke other products such as roll-your-own tobacco. A study by Siziya et al.^17^ demonstrated that the youths in the rural areas were more likely to smoke more than the youths in the urban areas owing to the accessibility of tobacco products mostly grown in the rural areas. Other factors could be at play but there is a need to investigate why youths in the rural areas smoke more than those in the urban areas. From the previous two GYTS done in Zambia 2002 and 2007, there has been no significant difference in the rates of current cigarette smokers^16,17,22,26^. This implies that despite existing interventions, the detrimental effects of adolescent smoking continue to pose a threat to a possible epidemic.

There is an association between age and cigarette smoking, the older adolescents are more likely to use tobacco use, this conforms with other studies^27^. Adolescents who had friends and parents who smoked were more likely to engage in smoking behavior than those who did not have friends or parents who smoked. This conforms with several studies that have found associations between friends and parents smoking status with current cigarette smoking status of adolescents^28,29^. The smoking behavior of an adolescent is related to the behavior of their parents and friends. There are different pathways the peers can influence smoking such as modeling of the risky behaviors and through normative peer pressure, however, the number of friends who smoke is the most common risk factor and more of a strong predictor than other peer influence factors^30^. In our study, we have demonstrated that as the number of friends who smoke increases, there is an increase in the odds of adolescents engaging in smoking behavior. This has been shown in other studies^30,31^. Our study has also shown that the smoking status of parents (father, mother, or both) is associated with adolescent smoking behavior. This was found in other studies^28,29^. The plausible explanation is that parental smoking status may be more of a direct parental influence than other parental measures, as this is related to parental rules at home, hence, creating a smoking environment for the adolescent^32^. Special consideration of parents smoking status should be taken into account when designing the adolescent behavior change intervention in Zambia.

In our study, slightly half of the students were taught in schools about the effects of smoking (53.6%) and the dangers of smoking (64.1%). The previous GTYS studies have shown that in 2002 in Lusaka, 46.6% of students had been taught in school on the dangers of smoking and 47.8% were taught on the effects of tobacco use. In 2007, only 48.3% and 49.5% of students had been taught in school on the dangers of smoking and the effects of tobacco use, respectively^26^. There is no substantial increase in students who responded being taught on the dangers and effects of smoking from 2002, 2007, and 2011 GTYS in Lusaka. Indeed, some rural areas such as Luangwa/Chongwe and Kafue also had equally low proportions of students being taught in schools on the dangers of smoking and the effects of tobacco use^26^. However, over 70% of Uganda and Kenyan students had been taught in schools on the dangers of smoking and the effects of tobacco use, which is a high level of exposure to health education on tobacco use^28^. After adjusting for age, sex, grade, and parents and friends smoking status, students who were not taught at school about the dangers and effects of smoking were more likely to smoke, although the association between smoking and being taught about effects of smoking did not reach statistical significance. This confirms previous studies that have not only shown positive and beneficial effects of the school-based curriculum but also the robust association between the curriculum and the change of adolescent behavior^33,34^. We expected that the students who had no discussions at school would be more likely to smoke, however, our study showed that students who had discussions on the dangers of smoking cigarettes were likely to smoke than those who had no discussion, this probably shows the ineffectiveness of the discussions to change the students’ attitude on cigarette smoking. This information calls for the development and implementation of an evidence-based school curriculum in all schools. Smoking in school-going adolescents is a complex problem, which needs multifaceted interventions such as legislation to make it difficult to access the tobacco products, parental supervision, and awareness of their behaviors, restriction of smoking on the school premises, but most importantly, schools should implement a standardized curriculum on smoking.

### Strength and limitations

Our results are subject to some limitations. Firstly, because of the observational design, we only were able to identify the factors associated with adolescent’s cigarette smoking and were unable to address causality. We were unable to assess changes in these factors over time. Despite adjusting for known confounders in the multivariable model, the potential for the residual confounders inherent in observational studies remains and might affect the interpretation of study outcomes. Secondly, the data used only represented the adolescents who were enrolled in school and who were present during the interview, this limits generalizability to all the adolescents in Zambia. However, the high school enrollment rate presents the possibility to generalize to all adolescents in the country. Also, a cross-sectional study of schools in Zambia has limited generalizability to other national or international settings. Thirdly, the data are based on self-reports, which might result in information bias related to misclassification of current cigarette smoking status. Malcon et al.^35^ demonstrated that there was evidence that the use of these smoking questionnaires had low sensitivity hence underestimating the tobacco use. Fourthly, we were not able to get data from the non-respondents thus selection bias could have occurred. The fifth limitation is the questionnaire does not capture other tobacco products to establish not only the cigarette smoking but also the smoking of other tobacco products among the school-going students. Overall response rate of the students surveyed was 55.7%, due to the low response rate the data were not weighted, posing a challenge in comparing the 2002 and 2007 GYTS data, which were weighted. Our study has the advantage of using the standardized questionnaire that enables the evaluation of smoking over time.

## CONCLUSIONS

When we compare the first and second waves of the GTYS study (2002 and 2007), the present study demonstrates that there has not been a substantial decline in cigarette smoking and there has not been an increase in the exposure of the health education on tobacco use among the school-going adolescents. The overall study provides additional evidence in support of the good school tobacco-related curriculum; it has demonstrated that there is an association between adolescent smoking behavior and the school tobacco-related curriculum. The curriculum alone would not reduce the rate of smoking, as it has to take into account relationships among parents, teachers, friends, and the tobacco regulatory laws, however, we can postulate that the implementation of a good and sustained tobacco school curriculum would bring a behavior change that would substantially reduce the rate of smoking among school-going adolescents in Zambia.

## Data Availability

The data supporting this research are available from the authors on reasonable request.
